# Antimicrobial peptides target ribosomes

**DOI:** 10.18632/oncotarget.4839

**Published:** 2015-07-12

**Authors:** Daniel N. Wilson, Gilles Guichard, C. Axel Innis

**Affiliations:** Institut Européen de Chimie et Biologie, Université de Bordeaux, Pessac, and INSERM U869, Bordeaux, France

**Keywords:** Chromosome Section, antimicrobial peptides, ribosome, protein synthesis, antibiotics

Antimicrobial peptides (AMPs) are a diverse group of molecules that play a vital role in the innate immune response of plants and animals [1]. With an average length of thirty or so residues, AMPs often feature a net positive charge due to a high arginine, lysine and/or histidine content. This in turn endows them with an amphiphilic character that enables them to associate with the phospholipid bilayer of bacteria, while staying clear from the eukaryotic cell membrane. It is no surprise then that many AMPs form transmembrane pores that disrupt the bacterial bilayer, causing rapid lysis and cell death. This lytic activity is a particular concern when it comes to developing AMP-based therapeutics, given that high peptide concentrations could ultimately result in unwanted cytotoxic effects on mammalian cells.

While pore-forming peptides account for a large fraction of known AMPs, other classes of peptides inhibit microbial growth by targeting intracellular processes, rather than through damage to the bacterial membrane. Among these, proline-rich antimicrobial peptides (PrAMPs) have attracted considerable attention in recent years as a possible way to counter the rapid increase in bacterial resistance to our dwindling arsenal of effective antibiotics. Contrary to the pore-forming AMPs, PrAMPs are transported into the bacterial cytoplasm by specific transporters, such as SbmA in Gram-negative bacteria. Such transport mechanisms are absent in mammalian cells and PrAMPS cross-react only mildly with intracellular eukaryotic proteins. As a result, they are generally considered to be non-toxic [2], making them ideal scaffolds upon which to develop novel alternatives to classical antibiotics. What's more, certain PrAMPs have been shown to cross the blood-brain barrier to selectively target brain cells, thus emphasizing their potential for tissue-specific drug delivery.

For many years, it was thought that insect-derived PrAMPs, such as drosocin, pyrrhocoricin or apidaecin, exert their inhibitory effects by targeting the bacterial heat shock protein DnaK and many PrAMP derivatives with increased affinity for this intracellular chaperone were developed as a result. Two recent papers, however, have challenged this view. Late in 2014, Krizsan *et al.* [3] showed that apidaecin and oncocin inhibit protein translation in bacteria by binding to and inactivating the 70S ribosome, a property that was shown to depend not only on cationic residues within the peptide, but also on a couple of conserved hydrophobic side chains. A month later, a study by Mardirossian *et al.* [4] noted that Bac7(1-35), a PrAMP featuring the N-terminal 35 residues of bovine cathelicidin Bac7, accumulates within *Escherichia coli* to high concentrations and inhibits protein synthesis by binding to the ribosome.

**Figure 1:**
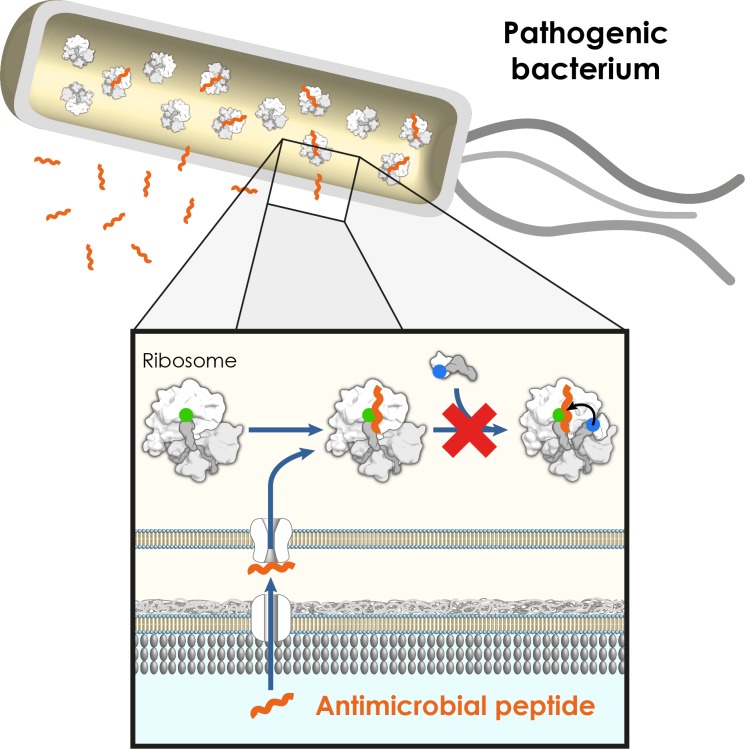
The antimicrobial peptide oncocin inhibits the transition from the initiation to the elongation phase of protein synthesis

Structural studies published by our groups (Seefeldt *et al.* 2015) [5] and by the Steitz laboratory (Roy *et al.* 2015) [6] further revealed the atomic details of the interactions between the bacterial ribosome and a 19-residue variant of the oncocin peptide, termed Onc112. Oncocin is produced by the milkweed bug (*Oncopeltus fasciatus*) and is representative of an entire family of PrAMPs, including pyrrhocorcin (firebug), metalnikowin (green shield bug), drosocin (fruit fly) and apidaecin (bee). The structures, and associated biochemical data, reveal that Onc112 interacts with the large subunit of the bacterial ribosome and blocks the binding site for an incoming aminoacyl-tRNA, thus effectively trapping the ribosome in an inactive initiation complex on the mRNA (Figure 1) [5,6]. Furthermore, this complex is itself destabilized due to probable steric clashes between the aminoacyl moiety of the initiator tRNA and the Onc112 peptide [5].

The binding site of Onc112 on the large ribosomal subunit overlaps with the binding site of many clinically important classes of antibiotics, such as the chloramphenicols, pleuromutilins, lincosamides and macrolides [7]. While this is very promising for the development of novel antibiotics, it is unclear whether resistance mutations that arise against currently used antibiotics will also confer cross-resistance against PrAMPs. The structural and biochemical knowledge acquired through these and further studies will therefore provide a solid framework upon which to design much-needed improved antibacterial compounds, either peptidic or peptidomimetic, that overcome multi-drug resistant pathogenic bacteria.
